# Evaluating Pay-It-Forward Strategy to Promote Hepatitis B Virus and Hepatitis C Virus Testing Among International Migrants From Low- and Middle-Income Countries in China: Protocol for a Cluster Randomized Controlled Trial

**DOI:** 10.2196/87165

**Published:** 2026-05-21

**Authors:** Mingzhou Xiong, Gifty Marley, Alikesi Muheyati, Mengqi Gao, Honghua Cao, Kimberly Enders, Shujie Huang, Joseph Tucker, Cheng Wang

**Affiliations:** 1 Institute for STDs/Leprosy Control and Prevention, Dermatology Hospital of Southern Medical University, No. 2 Lujing Road, Yuexiu District, Guangzhou, Guangdong, 510000, China, 86 13602463723; 2 Southern Medical University Institute for Global Health, No. 2 Lujing Road, Yuexiu District, Guangzhou City, Guangdong Province, Guangzhou, Guangdong, China; 3 University of North Carolina Project-China, Guangzhou, China, Guangzhou, Guangdong, China; 4 School of Public Health, Southern Medical University, Guangzhou, Guangdong, China; 5 Guangzhou Hengjian Hospital, Guangzhou, China, Guangzhou, Guangdong, China; 6 University of North Carolina at Chapel Hill, Chapel Hill, NC, United States; 7 London School of Hygiene and Tropical Medicine, London, England, United Kingdom

**Keywords:** pay-it-forward, hepatitis B, hepatitis C, testing, international migrants, low- and middle-income countries

## Abstract

**Background:**

Hepatitis B virus (HBV) and hepatitis C virus (HCV) are significant global health concerns, particularly prevalent in low- and middle-income countries (LMICs). In China, a significant number of international migrants from LMICs reside, many of whom are at high risk of HBV and HCV infection, while this group tends to engage in low HBV and HCV test use due to some adverse factors. Pay-it-forward (PIF) is a social innovation strategy that is based on the theories of upstream reciprocity and mutual aid. Previous studies have shown that the PIF strategy has proven effective in promoting sexually transmitted infections (STIs) testing in various populations.

**Objective:**

This study aims to evaluate the effectiveness of a PIF intervention in promoting HBV and HCV testing among international migrants from LMICs in China.

**Methods:**

A 2-arm cluster randomized controlled trial (RCT) will be conducted in Guangzhou, China. Participants will be recruited from a public hospital serving a large migrant community. A total of 100 eligible participants will be enrolled in blocks of 5 using a cluster randomization plan and randomly assigned to either the PIF intervention arm or the control arm in a 1:1 ratio. Participants in the intervention arm will watch a 2-minute video introducing the PIF concept and receive printed educational materials on HBV and HCV. Participants will then be offered free testing donated by previous participants, which they can accept or decline, and an opportunity to make a monetary donation to cover testing for a future participant. Control arm participants will receive standard medical services, with self-paid testing. The primary outcome is the proportion of participants tested for both HBV and HCV. Data will be collected through a self-administered questionnaire, and test information will be obtained from the hospital’s medical records without personally identifiable information. The survey data will be analyzed using generalized estimating equations to account for clustering effects.

**Results:**

This protocol was completed in August 2024, and implementation was conducted from September 2024 to February 2025. According to the baseline survey findings, 73.0% (73/100) of the 100 eligible participants recruited were male, 87.0% (87/100) were from African countries, and 85.0% (85/100) came to China for business purposes. About 40.0% (40/100) earned more than US $1100 per month, 62.0% (62/100) were married, and 6% (6/100) had both stable and casual sexual partners.

**Conclusions:**

This study is innovative in targeting international migrants from LMICs in China and using the PIF strategy to promote HBV and HCV testing. The PIF intervention is expected to increase testing rates by addressing financial barriers and fostering community support. The findings will contribute to the understanding of HBV and HCV testing promotion among this understudied population, with potential implications for public health policy and practice.

## Introduction

Hepatitis B virus (HBV) and hepatitis C virus (HCV) are both causes of acute and chronic disease and can be transmitted through unprotected sexual intercourse, infected blood, and mother-to-child transmission during pregnancy. Globally, the World Health Organization (WHO) estimates 254 million people have an HBV infection and 50 million people have an HCV infection, respectively [1,2]. Low- and middle-income countries (LMICs) experience a severe hepatitis burden, where the prevalence of HBV and HCV in the African and Western Pacific regions is higher than in other regions [3,4]. In China, persons with HBV or HCV make up 29.0% and 10.0% of all HBV and HCV cases globally [5]. Most people newly infected with HBV and HCV are often undiagnosed because the infection remains asymptomatic until decades after the infection, when severe symptoms develop with an increased risk of liver damage. Early diagnosis can help prevent the health problems associated with delayed diagnosis and onward transmission. The WHO recommends routine HBV and HCV testing for specific high-risk groups (including migrants from endemic regions) [1,2].

An international migrant is an individual who moves away from their usual country of residence, temporarily or permanently, for various reasons (excluding short-term travel and business trips across an international border) [6]. China’s Reform and Opening-up Policy has attracted many international migrants to live there. Over 846,000 registered international migrants lived in China by 2020, of whom 49.5% resided in Guangdong province [7]. About 95.0% of international migrants came from Asia, Africa, and Latin America, mostly from LMICs, including Vietnam, Brazil, and the Philippines [8]. Globally, international migrants bear a disproportionate burden of viral hepatitis, with migrant children and pregnant women being more vulnerable to infection risk [9]. Meta-analysis estimated that the pooled HBV surface antigen and antihepatitis B prevalence among international migrants were 4.4% (95% CI 3.4%‐5.5%) and 35.2% (95% CI 26.9%‐44.0%), respectively [10,11], and the pooled prevalence of HCV antibody (anti-HCV) and RNA (HCV-RNA) were 1.5% (95% CI 1.1%‐2.0%) and 0.6% (95% CI 0.4%‐0.9%), respectively [11]. Despite the high risk, various systemic barriers persist as global deterrents to health care use among international migrants. Among documented factors, language barriers significantly hinder HBV and HCV testing among migrants, limiting the capacity of health providers to offer testing and the willingness of migrants to accept testing. Additionally, institutional and technical factors, as well as religious issues, have been found to deter the use of health services among international migrants [12].

Findings from previous studies in China show that international migrants are generally young (average age 18‐49 years old), predominantly male, have a higher proportion of high-risk sexual behaviors, and are at an increased risk of hepatitis infection [12]. Factors like language and communication barriers, lack of knowledge of testing venues, lack of medical insurance, and low acculturation remain persistent barriers to the active uptake of HIV and STD testing services [13,14]. These factors also contribute to the reported low use of local medical services among international migrants in China [15]. Therefore, effective intervention measures are urgently needed to promote hepatitis testing uptake among international migrants in China to reduce the health burden and transmission rates [16]. These challenges have contributed to limited policy and financial support for health care promotion among international migrants, discouraging their complete integration into the local community in China and affecting their active engagement in these health care programs.

Pay-it-forward (PIF) is a social innovation strategy that has been proven effective in promoting health services uptake across various populations [17-19] and is based on the theories of upstream reciprocity and mutual aid [20-23]. In the PIF strategy, individuals receive a free test donated by others and are offered the opportunity to voluntarily donate to support another person anonymously [24]. Previous research in China has demonstrated that the PIF strategy can significantly increase STI testing uptake among high-risk populations, largely by leveraging active community engagement and peer-to-peer mobilization. Among men who have sex with men (MSM), gonorrhea or chlamydia and HBV or HCV testing rates are rising from 18.0% to 56.0% and 25.3% to 59.4%, respectively [17,19]. The PIF strategy also increased gonorrhea and chlamydia testing uptake from 4.0% to 82.0% among female sex workers in China [25]. Besides that, PIF has been successfully applied to promote vaccine uptake among vulnerable groups, such as older adults, children, and catch-up age girls [26,27]. Although these findings highlight the potential of PIF, its feasibility and effectiveness remain unexplored among international migrants in China, who face linguistic and cultural barriers and limited community mobilization for health initiatives. Thus, this research aims to evaluate the effectiveness of PIF in increasing HBV and HCV testing uptake among international migrants from LMICs in China using a cluster randomized trial.

## Methods

### Study Design

We will conduct a 2-arm, cluster randomized controlled trial (RCT) to evaluate the effectiveness and acceptability of a PIF intervention to increase HBV and HCV testing uptake compared with standard-of-care among international migrants from LMICs in China. Eligible participants who provide informed consent and complete the baseline survey will be assigned in a 1:1 ratio to one of the 2 study arms: PIF (intervention) or standard of care (control). Using a cluster-randomized design, each cluster of approximately 2–5 participants, defined as a group of patients that arrive together, will be randomly assigned to one of the 2 arms.

The cluster randomized design was selected for the following reasons: (1) to reduce the potential bias due to between-group contamination among participants who arrived with acquaintances or partners; (2) previous researchers found that participants are significantly more likely to receive HIV testing and donate for others when accompanied by a partner than when being alone; hence, the cluster randomized design can promote HBV and HCV test uptake and donation by peer influence; and (3) the cluster randomized design would simplify project management and improve adherence to intervention protocols more than an individual-based randomization [17,19]. We hypothesize that the PIF strategy will be superior to the control (standard medical services in the clinic) in increasing HBV and HCV testing among international migrants from LMICs in Guangzhou.

### Study Settings

This study will be conducted in Guangzhou, the capital of Guangdong province, China. Guangzhou is the primary trading destination for most international migrants in Southern China seeking greener pastures [28]. The interventions will be integrated into the standard procedures of a public hospital located in Sanyuanli, where the largest international migrant community from LMICs operates in Guangzhou [29]. This hospital was selected because it is dedicated and equipped to provide comprehensive health care services to the international population and is well patronized by the migrant community. The clinic is run by an experienced general medical practitioner who has engaged in health promotion among international migrants for over 10 years.

### Participant Recruitment

International migrants visiting the facility will be introduced to the study, and those who express interest will be screened for eligibility. After receiving the primary medical services for their visit, patients will receive co-created health education materials on HBV and HCV that provide basic information about HBV and HCV transmission routes, prevalence in China and among international migrants, prevention, and treatment. All educational materials to be used in the trial were co-designed by a co-creation group consisting of volunteer international students (n=4), an international migrant worker here for business (n=1), an international research fellow working in the field of sexual health in Guangzhou (n=1), a clinician from the international health clinic (n=1), and research staff (MX and AM; n=2). Eligible individuals willing to participate will be required to provide informed consent and complete the baseline survey.

Individuals who migrated to China from an LMIC, aged 18 years and older, have lived in China for at least one consecutive month, have not tested for HBV or HCV in the past 6 months, and were willing to provide informed consent will be eligible for inclusion in the study. Exclusion criteria include a self-reported previous chronic HBV and HCV diagnosis, unwillingness to provide informed consent, and inability to communicate in English, French, or Chinese.

Eligible individuals who provide informed consent will be redirected to the Wenjuanxing platform (Ranxing) to complete a self-administered survey. This baseline survey will collect data on participants’ demographic characteristics, sexual behaviors while in China, history of medical service use in China, and their knowledge of HBV and HCV before participation in the study.

### Randomization and Allocation

Clusters in this study are defined as small groups of participants who arrive at the clinic together or register within the same visit period and may therefore share information that could influence testing decisions. This definition is based on field observations that most international migrants arrive at the clinic alone or with a small number of companions (not more than 2 companions). Therefore, grouping all participants who arrive together into one cluster ensures the feasibility and validity of randomization with a minimum sample size. The decision to group eligible participants who arrive together into a cluster was based on field knowledge that most international migrants arrive at the clinic alone or with not more than 2 companions. Therefore, grouping all participants who arrive together into one cluster ensures the feasibility and validity of randomization with a minimum sample size. Once a cluster is formed, the entire cluster is randomized to either the PIF or control arm, following the pregenerated cluster randomization sequence created by an independent data analyst using SAS (version 9.4; SAS Institute). The allocation sequence will be implemented sequentially as clusters are enrolled. Only the independent data analysts will be blinded, as participants can determine their allocation based on the materials they receive.

### Intervention

Participants randomly assigned to the intervention arm will receive the PIF intervention. In the PIF arm, participants will receive counseling on HBV and HCV infection risks and the need for routine testing and HBV vaccination. They will then watch a 2-minute video (Multimedia Appendix 1) and receive a printed infographic card (Multimedia Appendix 2) introducing the PIF concept. The video and infographic card were drafted by the research team based on PIF research experiences among MSM and female sex workers and submitted to the co-creation group for review. The video introduced HBV and HCV, the risk of infection among international migrants, and the concept of the PIF strategy. The infographic card showed the four steps of PIF and provided the research WeChat (Tencent Holdings Ltd) QR code and a telephone number to facilitate participation. Only the revised versions of all materials approved by the co-creation group were used in the study.

Participants will be informed that they are eligible for a free HBV and HCV test, funded by donations from a previous participant. Testing will be voluntary, and participants consenting to testing will provide blood samples. Regardless of the testing decision, all participants will be offered the opportunity to make monetary donations of any amount to support testing for subsequent participants. Participants will be reminded that testing and donation choices are voluntary, and accepting or declining either or both will not disqualify them from the study. Donations can be made online by scanning the Alipay (Ant Group Co Ltd) or WeChat QR code provided or offline by depositing cash into the official PIF donation box placed in the physicians’ office.

Control arm participants will only receive the same educational materials on HBV and HCV, but no PIF-related information. They will be informed that they are eligible for HBV and HCV testing at an out-of-pocket cost of US $5.91 (43 RMB), as the test is not covered under insurance. Participants will be reminded that testing is voluntary and that they may decline without repercussions. Those who accept and pay the cost will be directed to provide blood samples for testing.

All tests in the study will be conducted using the HBV surface antigen test (Guangzhou Wondfo Biotech Co., Ltd) and the anti-HCV test (Guangzhou Wondfo Biotech Co., Ltd), which are approved for use in China and, at the time of this writing, used at the study site. Depending on their preferences, tested participants can receive their test results in person 2 hours after the sample provision or online via the phone number they provided as part of the hospital’s standard registration process. Those who test positive will be offered treatment services at the facility at their own cost. All participants in arms will also receive an informational card detailing the names, locations, working hours, and price lists of HBV vaccination centers near the study site.

### Outcomes and Measurement

The study’s primary outcome is dual HBV and HCV test uptake, defined as the proportion of participants who tested for HBV and HCV verified by hospital records. Secondary outcomes will include (1) HBV and HCV positivity rate, defined as the proportion of participants that tested positive; (2) donation rates and amounts, defined as the proportion of participants who donated and the total amount donated; and (3) cost-effectiveness analysis, defined as the test incremental cost-effectiveness ratio of the PIF model compared to the control arm. Prespecified subgroup analysis will compare HBV and HCV test uptake rates between PIF and the control arms by nationality, gender (male vs female), age (≤30 y vs >30 y), and level of education. Further details are summarized in Table 1 below.

**Table 1.** Primary and secondary outcomes with definitions.

Indexes	Definition
Primary outcome	
HBV^a^ and HCV^b^ test uptake	The proportion of participants who tested for HBV and HCV as verified by hospital records
Secondary outcome	
HBV and HCV positivity rate	The proportion of participants who tested positive
Donation rates and amounts	The proportion of participants who donated to others for HBV and HCV testing, and the total amount donated
Cost-effectiveness analysis	The test incremental cost-effectiveness ratio of the pay-it-forward (PIF) model compared to the control arm

a HBV: hepatitis B virus.

b HCV: hepatitis C virus.

### Sample Size

In a pretest study conducted from April 1 to May 27, 2024, among 20 international migrants from LMICs in Guangzhou, we observed a 90% (9/10) HBV and HCV test uptake rate in the PIF arm and 0% (0/10) in the control arm. However, we adopted the anticipated test rate from another study that applied the PIF strategy to promote HBV and HCV testing among MSM in China (59.4% in the intervention arm and 25.3% in the control arm) to ensure a stable result. Accordingly, we anticipated that 59.4% of participants would accept the dual HBV and HCV testing in the intervention arm and 25.3% of patients would accept testing in the control arm. Based on a 2-sided *t* test (unpooled), an intracluster correlation of 0.10, and an alpha of .05, an anticipated 10 clusters (consisting of approximately 5 participants per cluster) are needed in each intervention arm for the study to achieve an 80% power, resulting in a total sample size of 100 participants.

### Data Collection and Measures

**Figure 1.**
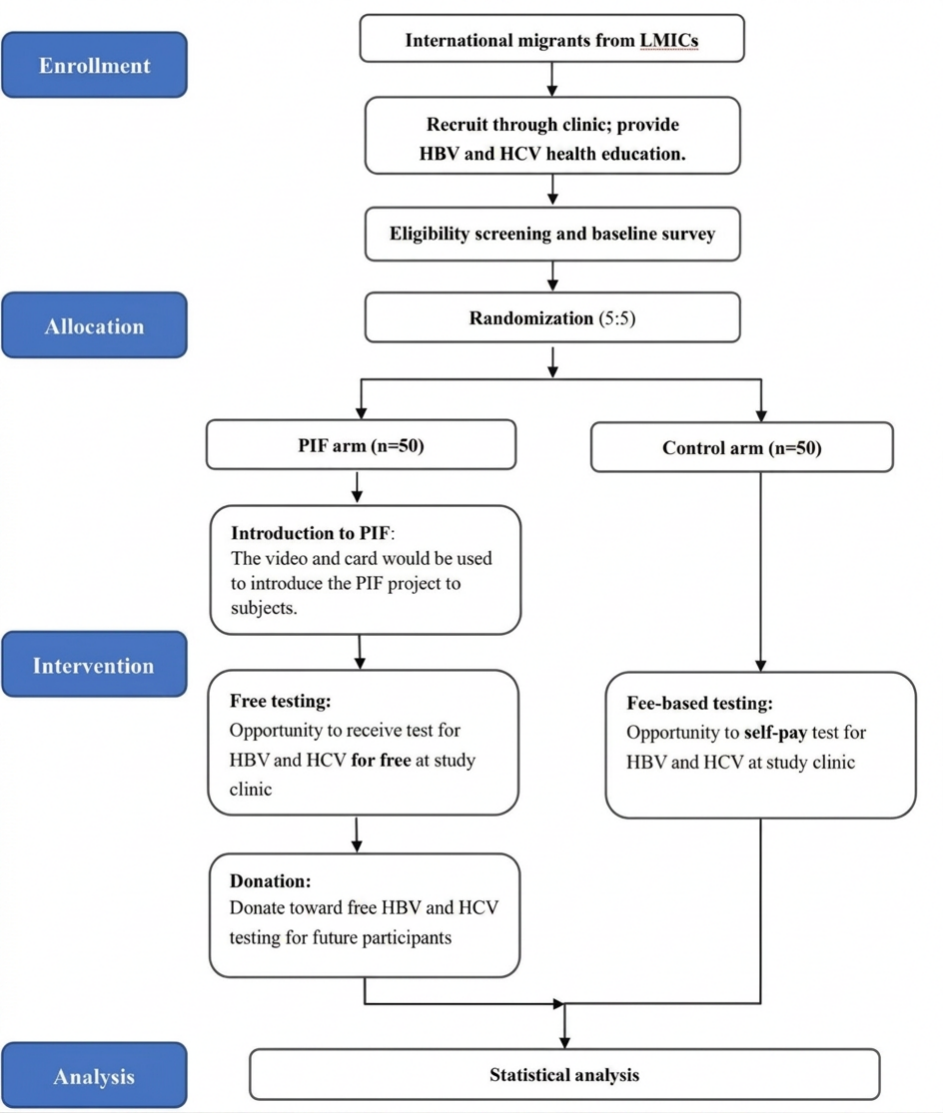
Flowchart showing the study design and recruitment process. HBV: hepatitis B virus; HCV: hepatitis C virus; LMIC: low- and middle-income country; PIF: pay-it-forward.

The study survey will be hosted on the Wenjuanxing platform (Ranxing), a web-based survey tool that meets industry standards for security and functionality [30]. The English, French, and Chinese versions of the questionnaire will be available to participants. To ensure questions are clear and culturally appropriate, the surveys were pretested with 5 English-native-speaking migrants, 5 Chinese natives, and 2 French-speaking migrants, who provided feedback used to revise the questionnaire and optimize the survey process (Figure 1).

The questionnaire includes four domains: (1) sociodemographic characteristics, (2) medical service use, (3) knowledge of HBV and HCV prevention, and (4) sexual behavior. Sociodemographic characteristics data will include age, country of origin, reasons for travel to China, level of education, monthly income after tax, and religious beliefs. Additional data will include previous chronic and transmissible disease diagnoses, medical service use experiences in China, and barriers to seeking health care in China. Seven questions will assess participants’ knowledge about HBV and HCV transmission, infection symptoms, HBV vaccine, and prevention measures. The survey will also collect data on participants’ sexual behaviors, like the number of regular and casual sexual partners, condom and drug use rates, and knowledge of the HBV and HCV status of sexual partners.

Participants can complete the surveys online by using WeChat or offline by completing the paper-based questionnaire. WeChat is a multipurpose social media smartphone app with messaging, multimedia, and financial functionality. There were over 1 billion monthly active users on WeChat in the first half of 2024 [31]. A project-dedicated iPad will also be provided for participants without a smartphone or WeChat account who opt to complete the questionnaire online. Printed copies of the survey will be provided to participants who prefer to answer offline. The physician and a research assistant will be available to assist participants and clarify questions as needed.

### Incentives and Reimbursement

Participants who complete the questionnaire will receive US $4.13 (30 RMB) as an incentive for their time. There will be no treatment cost reimbursements for participants who test positive for HBV and HCV and receive treatment at the study site. The free HBV and HCV test, valued at about US $5.91 (43 RMB), will only be provided to participants in the intervention arm.

### Data Analysis

Sociodemographic characteristics, sexual behavior, and HBV and HCV prevention knowledge data will be analyzed descriptively, and nominal variables will be tested using the *χ*^2^ test to compare differences between PIF and control group participants. The absolute proportions of dual HBV and HCV testing will be compared between the PIF and control groups using generalized estimating equations with a logit link, accounting for clustering effects. We will evaluate whether the PIF intervention is superior to the control arm in increasing HBV and HCV testing uptake rates by 20%. Multivariable models will adjust for potential confounders, including key sociodemographic factors and a previous STI diagnosis, selected a priori based on prior literature and theoretical relevance. Additional covariates associated with testing uptake in bivariate correlation (*P*<.20) will also be considered for inclusion in the final model to minimize residual confounding. Sensitivity analyses will also be conducted to assess the robustness of intervention effect estimates under alternative correlation structures, ensuring appropriate estimation even with modest cluster sizes. A complete-case analysis will be conducted if the primary outcome is missing for fewer than 15% of participants. If missingness in the primary outcome is ≥15% and determined to be random, multiple imputation will be applied.

### Ethical Considerations

Ethics approval was obtained from the Ethics Review Committee of the Dermatology Hospital of Southern Medical University (2014012), and written informed consent was obtained from all participants before enrollment. Personal information of all patients is required for HBV and HCV testing services at all hospitals in accordance with Chinese policy. This information will only be stored at the facility level and will be inaccessible to the research staff. All surveys are anonymous and do not collect any personal identifying information, such as name, passport number, or telephone number. The test results and questionnaire data will be linked through the unique project number. All survey data will be stored on a secure server at the Dermatology Hospital of SMU, accessible only with login information known to the research team. Participants who completed all the questionnaire surveys will receive US $4.13 (30 RMB) as an incentive for their time, but treatment costs will not be reimbursed or subsidized for those who tested positive and are linked to care.

### Monitoring

A data monitoring committee will not be formed for this RCT because the potential for harm to participants is minimal. Participants will be reminded that they can withdraw at any time if they experience an adverse event or undesirable effect from participating in this RCT. They will also be provided with the office contact of the principal investigator, CW, and the GDDH (Guangdong Province Dermatology Hospital) IRB (telephone: 020‐87255824) for reporting adverse effects and negative experiences. CW would be in charge of the interim analysis to evaluate implementation progress and quality and to decide whether to terminate or continue the trial. The research team members would access the interim data and results with the principal investigator’s authorization.

## Results

From June to July in 2024, a pilot survey was conducted, and the generated data informed the study’s standard operating procedures. A centralized in-person training session was organized for field workers, including 2 physicians and 1 nurse in the study clinic, 2 research assistants (undergraduate students), and 3 research team members (MX, AM, and GM) in August 2024. The project was officially launched at the end of August, and field recruitment of participants lasted from September 2, 2024, till February 12, 2025. The data generated from surveys were verified and cleaned from March to September 2025. The deidentified dataset was downloaded and analyzed using Microsoft Excel and R (R Core Team). Parts of the descriptive analysis were completed at the end of December 2025 (Table 1), and the study results are expected to be published before the end of 2026. This research has been funded by Guangdong Basic and Applied Basic Research Foundation (2022A1515110895) and Joint TDR/WPR Small Grants Scheme for Implementation Research in Infectious Diseases of Poverty (2021/109143).

A total of 138 international migrants from LMICs were recruited. Of this, 38 individuals declined participation due to (1) privacy concerns, (2) HBV and HCV testing in the past 6 months, (3) a current HBV infection, and (4) a language barrier. A total of 100 eligible participants provided informed consent and were enrolled in the study. Among them, 73.0% (73/100) were male, 62.0% (62/100) had ever been married, and 87.0% (87/100) were from African countries. About 56% (56/100) had only a regular sexual partner, 33% (33/100) were knowledgeable about HBV and HCV transmission routes, and 59.6% (42/71) were willing to get tested with a partner (See Table 2 below). PIF and control arm participants differed significantly in monthly income after tax (*P*=.03) and region of nationality (*P*=.03). Details of the testing uptake and the intervention effectiveness will be published separately.

**Table 2.** Descriptive summary of participant characteristics (N=100).

Variable	Pay-it-forward (n=50)	Control (n=50)	Total (n=100)	*P* value
Sex, n (%)				.50
Male	35 (70.0)	38 (76.0)	73 (73.0)	
Female	15 (30.0)	12 (24.0)	27 (27.0)	
Age (years), n (%)				.32
18‐35	13 (26.0)	16 (32.0)	29 (29.0)	
36‐45	19 (38.0)	22 (44.0)	41 (41.0)	
>45	18 (36.0)	12 (24.0)	30 (30.0)	
Marriage, n (%)				≥.99
Never married	19 (38.0)	19 (38.0)	38 (38.0)	
Ever married^a^	31 (62.0)	31 (62.0)	62 (62.0)	
Occupation, n (%)				.42
Business	41(82.0)	44 (88.0)	85 (85.0)	
Student	3 (6.0)	3 (6.0)	6 (6.0)	
No job	6 (12.0)	3 (6.0)	9 (9.0)	
Monthly income (US $)^b^, n (%)				.03^c^
<400	17 (34.0)	8 (16.0)	25 (25.0)	
400-1100	18 (36.0)	17 (34.0)	35 (35.0)	
>1100	15 (30.0)	25 (50.0)	40 (40.0)	
Education, n (%)				.29
High school and below	16 (32.0)	14 (28.0)	30 (30.0)	
Some college or vocational training	15 (30.0)	12 (24.0)	27 (27.0)	
Bachelor’s degree and above	19 (38.0)	24 (48.0)	43 (43.0)	
Regions, n (%)				.03^c^
Africa	44 (88.0)	43 (86.0)	87 (87.0)	
Southeast Asia	6 (12.0)	2 (4.0)	8 (8.0)	
South Asia	0 (0.0)	2 (4.0)	2 (2.0)	
Others	0 (0.0)	3 (6.0)	3 (3.0)	
Religious belief, n (%)				.20
Buddhism	3 (6.0)	2 (4.0)	5 (5.0)	
Islam	3 (6.0)	10 (20.0)	13 (13.0)	
Christianity	42 (84.0)	37 (74.0)	79 (79.0)	
Other^d^	2 (4.0)	1 (2.0)	3 (3.0)	
Sexual partners^g^, n (%)				.09
Regular partner only	22 (44.0)	34 (68.0)	56 (56.0)	
Casual partner only	6 (12.0)	3 (6.0)	9 (9.0)	
Both	5 (10.0)	1 (2.0)	6 (6.0)	
None	17 (34.0)	12 (24.0)	29 (29.0)	
Aware of HBV^e^ and HCV^f^ transmission routes^g^, n (%)				.67
Yes	15 (30.0)	18 (36.0)	33 (33.0)	
No	35 (70.0)	32 (64.0)	67 (67.0)	
Know the HBV and HCV status of their sexual partners, n (%)				.56
Yes	7 (21.0)	6 (16.0)	13 (18.0)	
No	26 (79.0)	32 (84.0)	58 (82.0)	
Willing to test for HBV and HCV with a partner, n (%)				.46
Yes	18 (55.0)	24 (63.0)	42 (59.6)	
No	15 (45.0)	14 (27.0)	29 (40.4)	

a Includes participants who were married, divorced, and widowed at the time of this writing.

b Exchange rate=7.17 yuan: 1 US $ at the time of the study.

c *P* value <.05 is statistically significant.

d Includes Hindu, Nature, Atheist, and non-religious/none.

e HBV: hepatitis B virus.

f HCV: hepatitis C virus.

g Variable recall duration limited to the past 6 months.

## Discussion

### Principal Findings

PIF is an intervention model shown to effectively increase health service uptake in China, particularly for STD prevention [19,25,27]. By combining subsidized services with community-driven funding, PIF integrates financial support with prosocial engagement, reducing costs while fostering community solidarity. Previous studies focused mainly on Chinese nationals and key populations like MSM in MSM-led clinics, limiting the generalizability of PIF to non-Chinese populations and its feasibility in public health care settings. This study aims to assess whether a standard PIF strategy can increase HBV and HCV testing among international migrants in China compared to standard care. The findings will provide empirical evidence of PIF’s adaptability to diverse populations and inform its potential integration into public clinics to enable broader implementation.

International migrants in China, particularly from LMICs, face significant, multifaceted barriers to health care use, including limited insurance coverage and economic constraints [14,15,32,33]. Previous research among international migrants from LMICs in China has shown high infection risks and prevalence of HIV and other STDs and a low willingness to use health services [13,15]. Despite the structural and social barriers, there has been little focus on promoting HIV and STD testing among these migrants. We anticipate that this PIF approach will increase HBV and HCV testing uptake beyond standard care and may inform policies to expand subsidized testing programs in China to include nonlocal populations.

International migrants from LMICs in China frequently demonstrate hesitancy to integrate into local culture and customs, which can strain relationships with the local Chinese population, and tend to seek greater self-identification with compatriots [32]. The PIF strategy can help participants feel empathy and support from fellow international migrants in a transmigrant city. Unlike HIV and syphilis, for which free testing can be obtained from China’s Voluntary Counseling and Testing (VCT) programs in most of the cities, hepatitis testing in China is unsubsidized and not covered by insurance. Thus, the out-of-pocket cost of testing reduces individuals’ willingness to get tested, especially if they are unaware of the infection risk. The PIF strategy provides a free testing opportunity based on hepatitis prevention education, which can increase their concern about the potential HBV and HCV infection risk and encourage them to get tested. These factors will ensure the anticipated effectiveness of the PIF strategy to promote HBV and HCV test uptake among participants.

The Chinese government has focused more on improving service availability and coverage for international immigrants since establishing the National Immigration Administration in 2018 [33]. However, health care facilities still face many economic-related barriers at the patient level when encouraging service uptake by international immigrants. The PIF strategy is an innovative and effective intervention measure in health services promotion, as it contributes to the subsidization of service costs through the donation of participants [17]. This cluster RCT is the first attempt to apply the PIF strategy in promoting HBV and HCV testing among international migrants. If successful, this strategy can be further adapted to the control of other sexually transmitted diseases, like chlamydia and gonorrhea testing, and promoting the uptake of prevention services, like vaccination, among international migrants in China.

This proposed study has several anticipated limitations. First, testing uptake will be measured at a single clinic in one city, potentially missing individuals who seek testing elsewhere, which may underestimate overall uptake and limit the findings for all migrants, particularly undocumented ones. However, the results will still aid in tailoring the PIF intervention for a larger RCT. Second, social desirability bias may affect self-reported sexual behaviors and health service use. To mitigate this, survey headers and research assistants will encourage honest responses. Third, donation amounts could be low, and the long-term sustainability of voluntary donations is uncertain. Future RCTs will assess the financial viability and explore strategies to enhance donation rates. Finally, this study will not include extended posttesting follow-up, so it cannot establish long-term uptake of linkage to care services. Participants who test positive for HBV and HCV will be offered treatment at their own cost, and exit interviews will investigate their reasons for accepting or declining treatment.

Our proposed study is innovative in both its target population and intervention mechanism, advancing evidence on strategies to promote HBV and HCV testing among international migrants from LMICs in China. Unlike conventional incentive-based models that rely exclusively on external subsidies, the PIF approach embeds voluntary donations within the service delivery framework, integrating financial facilitation with participatory engagement. This dual mechanism addresses both structural and psychosocial barriers to testing. By subsidizing out-of-pocket costs, PIF directly reduces the economic constraints that may deter testing uptake among uninsured migrants. Simultaneously, the donation component activates norms of reciprocity and collective responsibility, reinforcing existing mutual aid dynamics within migrant communities. The prosocial chain generated through this model may also enhance trust in the intervention and attenuate stigma associated with accessing testing services. This study extends prior PIF research beyond key populations and single-community settings, providing evidence on the scalability and contextual adaptability of community-participatory financing mechanisms in public health service delivery.

PIF is effective in leveraging upstream reciprocity and mutual aid to promote essential services delivery while spurring community solidarity to support marginalized users in the community. If successfully implemented among international migrants, PIF strategies could help mobilize micro-donations from financially better individuals to cover the cost of essential health care for affected financially challenged individuals.

### Future Directions and Dissemination

Results from this study will be disseminated through an open-access peer-reviewed publication and conference presentations. Project summary reports and policy briefs in English, French, and Chinese will be shared with the international migrant communities through their informal WeChat groups and printouts. The Chinese versions of the report and briefs will also be shared with the National Center for STD Control, the Chinese Center for Disease Control and Prevention, and the Guangdong Provincial Bureau of Disease Control and Prevention to inform STI programming and resource allocation for the province. The study findings will also inform the tailoring of the PIF to meet the contextual and cultural needs of the international migrant community in Guangdong province. The tailored PIF approach will be evaluated in a larger RCT across multiple clinic sites in multiple cities in Guangdong province.

## Supplementary material

10.2196/87165 Multimedia Appendix 1 A 2-minute video.

10.2196/87165 Multimedia Appendix 2 Infographic card.

## Abbreviations

GDDH: Guangdong Province Dermatology Hospital
HBV: hepatitis B virus
HCV: hepatitis C virus
LMIC: low- and middle-income country
MSM: men who have sex with men
PIF: pay-it-forward
RCT: randomized controlled trial
STD: sexually transmitted disease
STI: sexually transmitted infection
WHO: World Health Organization
