# Supplementary material for: Evaluating Pay-It-Forward Strategy to Promote Hepatitis B Virus and Hepatitis C Virus Testing Among International Migrants From Low- and Middle-Income Countries in China: Protocol for a Cluster Randomized Controlled Trial
Source: JMIR Res Protoc. 2026 May 21;15:e87165. doi: 10.2196/87165 (PMC13193702; doi:10.2196/87165)
Supplement: Multimedia Appendix 2 [file resprot-v15-e87165-s002.pdf]

Southern Medical University
Dermatology Hospital

Kindness
relay

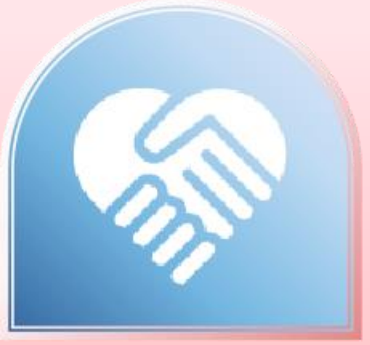

Pay-it-forward

Introduction

The Pay-it-forward project is a relay of kindness for foreigners in Guangzhou to get tested for hepatitis B and hepatitis C.

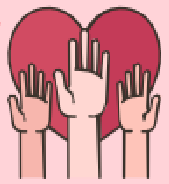

Foreigners provide donations to cover the cost of HBV and HCV testing for other foreigners in the community.

Today, you have received a free test from a donation by a foreign friend.

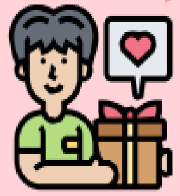

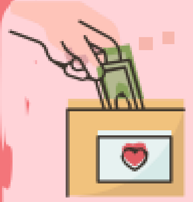

If you wish, you can also pass on the kindness by donating towards testing for the next person.

Your donation will benefit other foreign friends and give everyone a chance to live a healthy life.

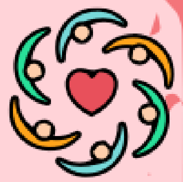

Channel

If you would like to join the Pay-it-forward study, please scan the QR code below（WeChat or Ali pay）：

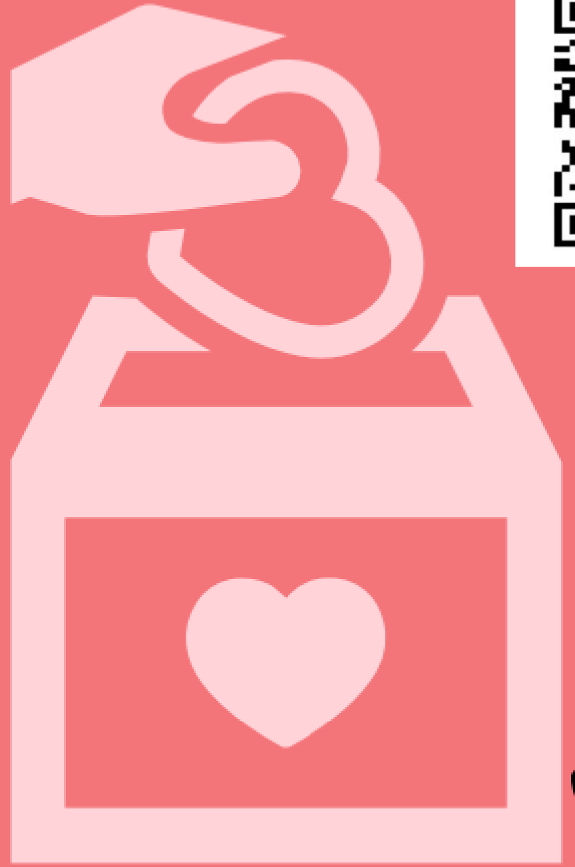

Every donation you make will help us provide free testing for foreign friends

Tel: 020 - 87255824

Thank you for joining us !

Contagious Kindness

to against hepatitis

Pay-it-forward
